# A New Treatment of Placenta Prævia

**Published:** 1848

**Authors:** H. S. Huber


### ARTICLE VI.

### *A New Treatment for some Cases of Placenta Prœvia.*

The following is an extract from a letter to me, from Dr. John Wiltbank, (Pr f. of Midwifery, etc, in the Med. Department of Penn. College, at Phila.,) formerly my preceptor.

After giving me a history oi the case from seven and a half months, when the flooding commenced, and the means employed to control it, up to the completion of gestation, he thus continues.

"On the 2nd of July, about 9 o'clock, A.M., I saw her.

The pains were regular and tolerably frequent, a gush of blood attended every pain. Upon examination, the os was soft and somewhat dilated, and the placenta plainly felt surrounding it, blocking it up and preventing my feeling the child.

"I sat by the patient watching her with much anxiety. My plan was, to wait for the dilatation, and then to turn and deliver by the feet. At 12 M., I proceeded to act. She had lost considerable blood, and the parts were pretty well dilated. I judged from the action of the fœtal heart that the child was living; and in the first presentation of the vertex. I therefore bared my left arm, introduced my hand into the vagina, then, very cautiously into the uterus, separating the placenta at the posterior right side, and ruptured the membranes.

"I found the head low down in the pelvis, so that I could neither pass my hand, nor push up the head, without risk; at the same time the flooding was tremendous, and the patient sinking. It was a moment of great perplexity; *I closed my hand, and with the back of my fist pressed firmly upon the (inside of the) perineum.* This brought on a powerful bearing pain, which in a moment forced the head thr ugh the side of the placenta, upon my fist, and both the head and fist through the soft parts. Another pain delivered the rest of the child; the placenta soon followed, the flooding ceased, and both mother and child did well. Thus terminated most happily a case that caused me much anxiety. The lady is still anemic, but is improving daily.

"Pressure upon the perineum, in such a case, is new practice, suggested at the moment by difficulties by which I was surrounded, but I should think it may be found in other cases the best way of managing them." During my pupilage, I was taught by Dr. W. that pressure on the inside of the perineum, when the pains were feeble, or at very long intervals, during the latter stage of natural labor, would increase their intensity and frequency, and I have often employed it for that purpose; pressure, then, with the palmar surface of the fingers sufficing. But we here have a new application of the principle that such pressure will stimulate the uterus to forcible contractions, and in a situation where we require all the resources of our art.

The above extract is from a familiar letter, not intended for publication, and which I have thus used without the Doctor's knowledge. H. S. HUBER.

